# The initiation and maintenance of gonadotropin-releasing hormone neuron identity in congenital hypogonadotropic hypogonadism

**DOI:** 10.3389/fendo.2023.1166132

**Published:** 2023-04-27

**Authors:** Wilson CJ Chung, Pei-San Tsai

**Affiliations:** ^1^ Department of Biological Sciences, Kent State University, Kent, OH, United States; ^2^ Department of Integrative Physiology, University of Colorado, Boulder, CO, United States

**Keywords:** gonadotropin-releasing hormone, congenital hypogonadotropic hypogonadism, neurogenesis, neuronal de-differentiation, epigenetic factors, environmental factors

## Abstract

Neurons that secrete gonadotropin-releasing hormone (GnRH) drive vertebrate reproduction. Genetic lesions that disrupt these neurons in humans lead to congenital hypogonadotropic hypogonadism (CHH) and reproductive failure. Studies on CHH have largely focused on the disruption of prenatal GnRH neuronal migration and postnatal GnRH secretory activity. However, recent evidence suggests a need to also focus on how GnRH neurons initiate and maintain their identity during prenatal and postnatal periods. This review will provide a brief overview of what is known about these processes and several gaps in our knowledge, with an emphasis on how disruption of GnRH neuronal identity can lead to CHH phenotypes.

## Introduction

Gonadotropin-releasing hormone (GnRH) secreted from GnRH neurons drives sexual maturation and reproduction in all vertebrates. GnRH neurons develop prenatally *via* a complex multi-step process that establishes their connectivity and differentiation state, ultimately allowing them to synthesize and release GnRH (1). Following their development, the continued maintenance of GnRH neurons is required for puberty and a healthy reproductive lifespan. Much of studies on GnRH neurons have focused on specific stages of their development and the control of their postnatal secretory activity, but significant gaps in our knowledge still exist regarding multiple aspects of GnRH neuronal biology. Efforts to fill these knowledge gaps are further complicated by the heterogeneity of the GnRH system that gives rise to subpopulations of GnRH neurons with seemingly different biological properties and responsiveness to signaling molecules (2).

In past decades, a large number of genes causally linked to GnRH deficiency have been uncovered in humans and rodents (3). Mutations in many of these gene in humans often lead to congenital hypogonadotropic hypogonadism (CHH), a secondary form of hypogonadism characterized by low circulating gonadal steroids, partial or absent puberty, and infertility (4, 5). Although not immediately life-threatening, CHH deleteriously and chronically impacts fertility, mental health, bone growth, and metabolism (6, 7) in ways that severely erode the patients’ quality of life. A large number of CHH-related genes are thought to control (1) prenatal GnRH neuronal migration or (2) postnatal GnRH secretion. However, a closer inspection reveals an additional need to consider genes and epigenetic factors that drive *GnRH* expression during prenatal and postnatal periods to ensure GnRH neuronal identity. This review aims to more specifically discuss the initiation and maintenance of GnRH neuronal identity during prenatal neurogenesis and postnatal maintenance periods with an emphasis on how the disruption of these processes can lead to reproductive disruption similar to the CHH phenotype.

## The developmental stages of the mammalian GnRH system

GnRH neuronal development consists of three major phases: (1) neurogenesis to initiate GnRH neuronal identity, (2) migration to the forebrain, and (2) extension of axons to the median eminence. Studies in mice have provided much insight into these three phases. Using immunocytochemistry, *in situ* hybridization and birth dating techniques, two landmark mouse studies found the earliest *GnRH*-expressing neurons on embryonic day (E)11.5 in the medial ventral olfactory placode (OP) at the tip of the nose, from where they migrate into the forebrain (8, 9). The majority (i.e., ~80%) of mouse GnRH neurons undergo their last division between E9.5 and E10.5 (8). Taken together, the period of GnRH neuron neurogenesis has been defined to occur between E9.5 - E11.5. Following this neurogenesis phase, GnRH neurons enter a migratory phase in which they follow the olfactory, vomeronasal and terminal nerves through the cribriform plate to enter the preoptic area and hypothalamus. After the cessation of migration, GnRH neurons extend and target their axons to the external zone of the median eminence for GnRH release (10). In mice, the formation of the GnRH system is largely complete around E16.5 (9, 11–17). Postnatally, most GnRH neuronal cell bodies reside near the anterior tip of the third ventricle called organum vasculosum lamina terminalis in the preoptic area (1, 18–20). Of comparative importance, the GnRH system develops similarly in humans and non-human primates, but with additional GnRH neurons found in the tuberal hypothalamus (21–23).

## CHH and Kallmann syndrome

Human and mouse studies have significantly advanced our understanding of molecular control of GnRH neuronal development and function in health and disease. Many studies have focused on understanding the pathogenesis of Kallmann syndrome (KS), a subset of CHH caused by heritable GnRH deficiency. KS has all the clinical presentations of CHH, including absent puberty and infertility, but with an additional phenotype of anosmia (5, 24). KS is believed to be caused primarily by developmental defects in GnRH neurons, whereas normosmic CHH may have additional causes involving pituitary or kisspeptin system defects. A landmark study conducted in a human fetus with KS reported GnRH neuronal migration was impaired when the gene *ANOS1* was mutated. In this case, GnRH neurons were arrested anterior to the cribriform plate, just outside the brain, rather than arriving at their normal destinations in the preoptic and hypothalamic regions (23). Later studies in humans and rodents support that defects in GnRH neuronal migration is a major cause of CHH (5, 24). However, KS, as well as CHH, are highly heterogeneous in their causes and phenotypes. Increasing evidence suggests KS and CHH gene mutations may also disrupt the neurogenesis of GnRH neurons during development (1, 12, 25–36). Of note, the disruption of GnRH neuron neurogenesis has, thus far, received much less attention than GnRH neuronal migration in the context of KS or CHH.

## Neurogenesis of GnRH neurons

The anatomical location of nascent GnRH neurons in various animal models suggests GnRH progenitor cells are likely derived in the embryonic medial-ventral OP. However, recent cell lineage tracing studies show a more complicated picture. The OP is an ectodermally derived structure that gives rise to both non-sensory respiratory epithelium and sensory olfactory epithelium. The olfactory epithelium eventually develops into the main olfactory and vomeronasal systems (37, 38). Ablation studies in rat and chick embryos suggest a small percentage of GnRH neurons may have come from the respiratory epithelium, whereas the majority of GnRH neurons are derived from the olfactory epithelium (39–44). Studies in mice similarly show that GnRH progenitor cells are not exclusively localized in the olfactory epithelium, but can also be detected in the respiratory epithelium by the expression of a *transcription factor activator protein 2α* (*TFAP2α*) (45). In the same study, a number of GnRH neurons are found to be positive for nestin, another marker for the respiratory epithelium. Lastly, some GnRH neurons do not express olfactory epithelium markers, such as *Mash-1*, *Math4C*/*neurogenin1*, and *NeuroD* (45). Taken together, these data suggest that both the respiratory and olfactory epithelium contribute to the GnRH progenitor pool in the OP.

In several vertebrate species, the neural crest has been implicated as a possible origin of GnRH progenitor cells (46–48). The anterior neural crest, which arises from the lateral edge of the neural plate, shares a border with the region that eventually becomes the OP. Neural crest cells have been shown to migrate into the presumptive OP, and therefore are likely to have contributed to cell populations in the developing OP (49). This notion is supported by studies in zebrafish demonstrating a fraction of neural crest cells labeled before their migration are later found to express *GnRH* mRNA and peptide (50–52). In mice, a small proportion (i.e., ~30%) of GnRH neurons found in the OP also have a genetic lineage similar to *Wnt1-* and/or *Islet1/2*-expressing progenitor cells that characteristically arise in the neural crest (47, 53), but the remaining 70% GnRH neurons are thought to have a placodal ectodermal origin instead of a neural crest origin (2). Taken together, GnRH neurons are currently thought to have a dual origin of both the neural crest and OP (2). This could explain, in part, the heterogeneity of GnRH neurons and why many gene knockout studies are only able to ablate a fraction of GnRH neurons (54).

Recent advances in genetic screening have identified many causal genes for CHH. There is an extensive list of up to 54 CHH causal genes according to one recent review (3). Of these, eight genes (*Fgf8*, *Fgfr1, Fgf17*, *Dusp6*, *Flrt3*, *Il17rd*, *Klb* and *Spry4*) are associated with fibroblast growth factor (Fgf) signaling either as a ligand, receptor, co-receptor, or synexpression gene. All genes except *Spry4* and *Klb* are thought to be involved in the neurogenesis of GnRH neurons (3, 28, 55). The current consensus is that Fgf8, a ligand in the Fgf signaling family (56), acts as an upstream Fgf signaling factor to control the neurogenesis of GnRH neurons. Studies in humans and rodents have cemented the concept that Fgf signaling is critical for the neurogenesis of GnRH progenitor cell birth and proliferation (1, 26, 28, 32). As described above, some CHH/KS patients harbor mutations in the *Fgf8* or *Fgfr1* gene (28, 57). In fact, homozygous *Fgf8* hypomorphic mice exhibited a complete loss of GnRH neurons as early as E11.5, at the time of their emergence, suggesting GnRH neurons did not undergo normal neurogenesis when *Fgf8* was deficient (12, 28). Further, *Fgfr1* hypomorphic newborn mice showed a similar reduction in the GnRH system (i.e., ~90%) (12), suggesting Fgf8 may act through one of its cognate receptors, Fgfr1, to support neurogenesis of GnRH neurons. Heterozygous *Fgf8* hypomorphic mice suffered a 50% reduction in GnRH neurons but did not exhibit any migratory defect (12). Taken together, reduced Fgf8/Fgfr1 signaling caused failed neurogenesis of GnRH neuron rather than GnRH neuron migration. Later studies reported that the reduced GnRH neuronal system in these hypomorphic mice caused abnormal reproductive function (58). These results not only provided a fundamental explanation for the reproductive defects found in KS/CHH patients who harbor *Fgf8/Fgfr1* mutations, they also suggested the initial neurogenesis of GnRH neurons was highly dependent on *Fgf8* expression level. The four other CHH causal genes associated with Fgf signaling and thought to influence neurogenesis (*Fgf17*, *Dusp6*, *Flrt3*, and *Il17rd*) are classified as *Fgf8* synexpression genes that likely modulate Fgf8 activity during development (55).

Currently, it is unknown whether *Fgf8* deficiency leads to the elimination of the GnRH progenitor cells by abrogating cell proliferation or cell survival. Circumstantial evidence favors the second possibility given that increased apoptosis has been reported for the E10.5 medial-ventral OP when *Fgf8* is deficient (1). Because cell lineage studies confirmed that *Fgf8* mRNA expression is primarily localized in the respiratory epithelium (59), we posit that *Fgf8-*expressing respiratory epithelial cells secrete Fgf8 to provide trophic support for the survival of GnRH progenitors in the OP. In addition, fate specification studies in human pluripotent stem cells show that Fgf8 is also required to program these stem cells towards GnRH neuronal fate (60, 61). That said, it is also possible that newly emerged GnRH neurons may be vulnerable to cell death and require Fgf signaling for survival. An early report indicated that in mice, the number of GnRH neurons peaked to 2000 at E12.75 but were “pruned” by almost 50% around the time of birth (62). GnRH neuron-specific deletion of *neuropilin 1* (*Nrp1*), the cognate receptor for the repulsive guidance cue semaphorin 3, resulted in excess prenatal and postnatal GnRH neurons, suggesting a defect in this pruning (63). Of interest, Fgfr1 has been shown to interact with Nrp1 (64) and may be involved in this process.

Overall, the literature suggests Fgf8/Fgfr1 signaling is critical for the survival of GnRH progenitor cells and their differentiation into GnRH neurons. However, a possible role of Fgf signaling in the survival of newly emerged GnRH neurons cannot be ruled out.

## The maintenance of postnatal GnRH neurons

The prenatal development of GnRH neurons is a well-studied process with decades of insights (65). In contrast, the postnatal maintenance of GnRH neurons remains poorly understood despite its physiological significance. Understanding the maintenance of the postnatal GnRH system also imparts significant translational value because postnatal deficits in the nervous system typically occur after the critical developmental period and can theoretically be reversed with proper stimuli. This plasticity, if better understood, could be harnessed for the treatment of fertility issues in humans and other animals.

For this section, we will endeavor to address two questions. First, can a disease state such as CHH lead to the postnatal loss of fully differentiated GnRH neurons? Second, can the postnatal GnRH neuronal loss be reversed by beneficial cues? In the context of this discussion, we broadly define two mechanisms of postnatal GnRH neuronal loss as either neuronal death or the loss of GnRH neuronal identity (de-differentiation). Since the only unifying marker for differentiated GnRH neurons is GnRH itself, the long-term loss of GnRH production is viewed as neuronal de-differentiation (66).

We first ask the question if a disease state can lead to the postnatal loss of differentiated GnRH neurons without impacting their development. One interesting observation from rodent and human studies is that the number of postnatal GnRH neurons in the hypothalamic/preoptic area of healthy individuals remains largely unchanged within a broad age range examined (22, 33, 67, 68), leading to the assumption that once a mature GnRH system is established, it continues to persist during the animal’s lifespan. However, this assumption may not be true. Several lines of evidence suggest the postnatal GnRH system is indeed vulnerable to disruption and can be lost under certain disease states. The first evidence came from a mouse model of Huntington’s disease, which was born with a normal set of GnRH neurons but lost 90% of them by 12 weeks of age (69). The second evidence came from a transgenic mouse model expressing a dominant negative Fgf receptor (dnFGFR) in GnRH neurons. The expression of this transgene interferes with the function of two endogenous Fgf receptors (Fgfr1 and Fgfr3) specifically in GnRH neurons (35), resulting in GnRH neurons with reduced responsiveness to Fgf signaling. The dnFGFR mice had normal GnRH neuron number at birth but underwent a progressive GnRH neuronal loss as they aged, losing up to 70% of their GnRH neurons by 550 days of age (33). Parallel to their postnatal GnRH neuronal loss, dnFGFR mice experienced accelerated reproductive senescence (35). In a similar vein, mouse models of Down syndrome (70) and *Fgfr3* deficiency (26) were both born with normal number of GnRH neurons but exhibited a significant loss of postnatal GnRH neurons in adulthood. It is currently unclear if GnRH neuronal loss from these studies resulted from cell death or de-differentiation.

Although postnatal GnRH neurons have previously been suggested to undergo apoptotic death following exposure to toxins (71, 72), death as a cause of postnatal GnRH neuronal loss has never been documented in the whole organism. More indirect evidence suggests postnatal GnRH neurons may, more frequently, undergo de-differentiation and completely lose their ability to produce GnRH. Evidence for this notion again came from studies on dnFGFR mice (33, 35). The reduced GnRH neurons and lower fertility in dnFGFR mice could, later in life, be restored back to normal by cohabitation with an opposite-sex (OS) cage mate, suggesting their GnRH neurons were not dead but instead temporarily de-differentiated and had the potential to return to normal activity. Supporting this possibility, lineage tracing studies showed that the conditional deletion of a transcription factor *Vax1* (ventral anterior homeobox 1) in GnRH neurons rendered GnRH neurons unable to produce GnRH without impacting their survival (73). In addition, approximately 10% of CHH patients experienced spontaneous reversal to gain fertility after the termination of hormone replacement therapy (74–79). These patients included those harboring mutations on genes such as *Fgfr1*, *Chd7*, *Prokr2* originally thought to irreversibly disrupt GnRH neuronal development and reduce postnatal GnRH neuronal population. These findings collectively suggest some GnRH neurons in these patients may be originally dormant and de-differentiated due to genetic lesions, but later became reactivated in life to drive the reproductive axis.

If de-differentiated GnRH neurons could be recovered, an important question would be: what type of signals can stimulate this recovery in the postnatal GnRH system? Robust postnatal plasticity in the GnRH system has been observed during puberty in multiple forms (80), including but not limited to the expression of receptor for a major GnRH stimulator, kisspeptin (81), firing properties (82, 83), and morphology (84–86). In this respect, the postnatal GnRH system remains plastic and may have an inherent resilience to recover from deficits generated during development and from a disease state.

**Figure 1:**
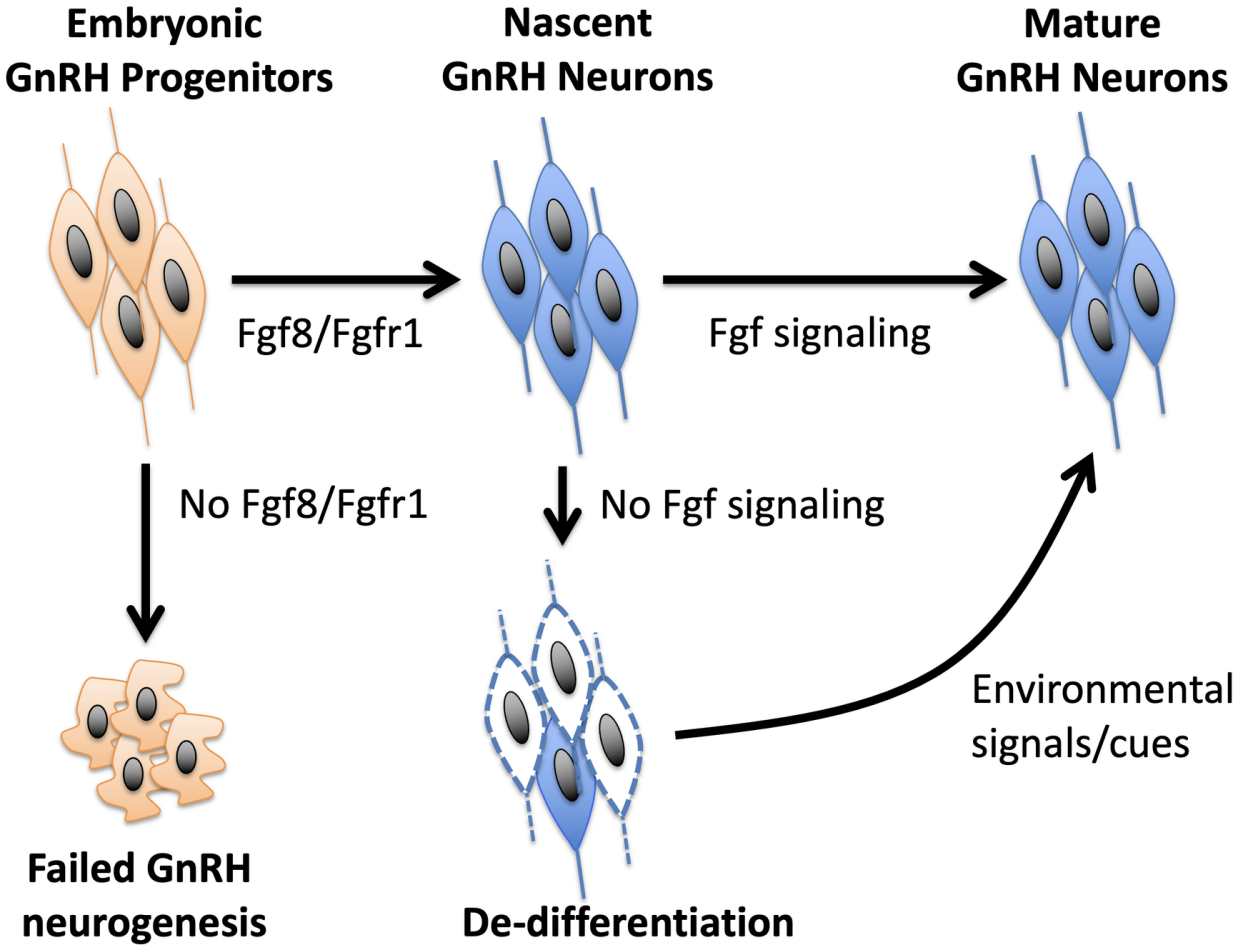
A model of how Fgf signaling controls the neurogenesis and differentiation of GnRH neurons. Fgf8 signaling through Fgfr1 is critical for GnRH neurogenesis from GnRH progenitors. Once nascent GnRH neurons are formed, continued exposure to Fgf signaling is required to maintain their differentiated state. In the absence of Fgf signaling, GnRH neurons fail to express *GnRH* and become de-differentiated. However, this de-differentiated state can be reversed by positive environmental signals, such as OS housing, that may upregulate neuroprotective molecules in the brain.

Although the nature of specific cues that could restore a de-differentiated postnatal GnRH system is poorly understood, some insight could be gained from the dnFGFR mouse model (33, 35). Because the declining GnRH system and reproductive function were restored back to normal in dnFGFR mice housed with an OS, but not same sex (SS), partner (33), an intriguing hypothesis is that the postnatal reproductive brain, like the cognitive brain, is highly plastic and responsive to environmental cues, such as olfaction It is possible that OS housing in mice represents one of many forms of environmental enrichment that may alter signaling in the brain to restore the GnRH system and fertility. Indeed, the GnRH system of several vertebrates can respond acutely to environmental stimuli with a strong socio-sexual context (87–91). For example, cichlid fish showed a rapid increase in *GnRH* expression in response to improved social status (90), and breeding stimuli increased the number of GnRH neurons in amphibians (87) and birds (89, 91). In the musk shrew, the exposure of prepubertal females to males increased the number of GnRH neurons (88). In mice, pheromones serve as important signals that may accelerate (92, 93) or inhibit (94, 95) pubertal onset. The secretion of luteinizing hormone (LH) and testosterone (T) in male mice can also be triggered acutely by pheromones from female mice (96–101). Although humans do not exhibit pheromonal sensitivity comparable to other mammals, LH and T secretion in humans can also be acutely activated by OS interactions or sexually arousing visual cues (102–104). Perhaps OS exposure generates a combination of sensory and social cues that work in concert to upregulate protective signaling molecules in the brain capable of reversing the de-differentiated state of GnRH neurons. Identifying these cues will provide insights into the general nature of environmental signals to adopt or avoid for fertility improvement. These insights may broadly benefit fertility in individuals with a compromised GnRH system resulting from postnatal GnRH neuronal de-differentiation. A model summarizing the involvement of Fgf signaling and OS housing in the development and differentiation of GnRH neurons is presented in **Figure 1**.

## Epigenetic control of prenatal and postnatal GnRH neurons

Increasing evidence suggests *GnRH* gene expression, thus GnRH neuronal identity, is controlled by epigenetic mechanisms during both prenatal and postnatal periods (105–107). Importantly, environmental stimuli can epigenetically alter the expression of genes critical for cellular processes, such as cell proliferation and fate specification, to affect long-range transcriptional interactions with enhancer or silencer regulatory sequences (108). In general, definitions that describe epigenetics range from those that include heritable gene function to those stating that epigenetic changes are molecular events that remodel chromatin without altering the primary genetic code (109). To initiate and maintain the identity of GnRH neurons, the *GnRH* promoter must be active. The *GnRH* promoter has been shown to undergo major chromatin and methylation changes that resulted in the loss of their ability to produce GnRH (110). For example, *GnRH* promoter elements displayed higher sensitivity to DNAseI treatment, indicative of more accessible chromatin, in mature GT1-7 GnRH neuronal cell line compared to the immature GN11 GnRH neuronal cell line (110). These GnRH regulatory elements also displayed RNA polymerase II enrichment as well as the active histone marker, H3K4me3, indicating that maturation of GnRH neurons relied on chromatin changes on GnRH regulatory elements (110). Indeed, one of the earliest studies of epigenetic regulation of GnRH neuronal identity was found in Rhesus macaque monkeys (111). *GnRH* gene demethylation was followed by a rise in *GnRH* mRNA expression, indicating that the epigenetic events were critical for *GnRH* promoter activation. Interestingly, a similar epigenetic mechanism drove pubertal onset (111). Studies showed that GnRH gene demethylation leading to *GnRH* expression may have been a Ten-Eleven Translocation (TET)-dependent process. The TET family consists of three isoforms (*Tet1, Tet2, Tet3*) which demethylate CpG dinucleotides to 5-hydroxymethyl (5hmC), 5-formyl (5fC), and 5-carboxyl (5caC) (112). Loss of TET proteins decreased overall gene expression and demethylation at promoters of active genes (113, 114). In 2016, Kurian et al. found that *Tet2* overexpression could increase *GnRH* mRNA by altering H3K4me3 abundance associated with the *GnRH* promoter (115). Moreover, GnRH-specific *Tet2* knockout mice displayed lower plasma LH levels and had lower fecundity in adult males, indicating an involvement of epigenetic factors in GnRH neuronal function (115). Lastly, in polycystic ovarian syndrome (PCOS) patients with GnRH neuron hyperactivity, *Tet1* was hypomethylated (over-expressed), further suggesting the importance of DNA methylation in controlling GnRH promoter activity and possibly maintaining GnRH neuronal identity (116).

Importantly, effects of environmental stimuli on the epigenetic may not only disrupt GnRH promoter activity but may also affect the transcriptional control of genes (i.e., *Fgf8*) required for GnRH neuron neurogenesis. For example, recent studies showed that in the olfactory region, *Fgf8* transcription during the neurogenesis phase of GnRH neurons is under DNA methyltransferases (DNMT) and TET1 control (31, 117, 118). Moreover, there is evidence supporting that TET function is not limited to its enzymatic activity to catalyze CpG demethylation (112, 119), but also its ability to recruit EZH2, a polycomb repression complex 2 protein, which catalyzes H3K27 methylation. Indeed, elevated H3K27me3 association with the mouse OP *Fgf8* promoter coincided with EZH2 enrichment (31). These data led to the hypothesis that the presence of TET1 on the *Fgf8* promoter not only catalyzed its demethylation, but also functioned as a molecular anchor for the recruitment of epigenetic factors to facilitate H3K27me3 marks and repress gene transcription (112, 119).

Lastly, epigenetic control has been reported in postnatal peripubertal female rats exposed to DNMT inhibitor, azacitidine, which exhibited smaller ovaries and underwent delayed puberty (120, 121), indicating that abnormal demethylation disrupts reproductive health (120). Similarly, recent studies found that microRNA-mediated epigenetic changes contribute to the timing of pubertal onset (122). These results imply that epigenetic factors not only critically control prenatal GnRH neuron development, they could also mediate the maintenance of GnRH neuronal identity during postnatal GnRH neuron maturation and function.

## Conclusions and perspectives

Recent advances in whole genome and exome sequencing have facilitated the identification of genes associated with CHH and KS. These genes, when validated in animal models, provide excellent mechanistic insights into the development and function of the GnRH system. That said, CHH and KS are highly heterogeneous disorders with diverse etiologies beyond the frequently described disruption in GnRH neuronal migration or secretion. Disrupted neurogenesis of GnRH neurons is one of the less described etiologies. In addition, even genetically identical probands with the same mutations in CHH causal genes could have very different clinical presentations (34), suggesting an involvement of environmental influence and epigenetic control. As such, genetic and epigenetic factors that drive GnRH neurons to initiate and maintain their identity is an underexplored area and deserve greater attention in the context of CHH and KS.

## Author contributions

WC contributed to the research and writing of the developmental and epigenetic sections, and P-ST contributed to the research and writing of the postnatal, introductory, and conclusion sections. Both authors contributed equally to the integration of the ideas. All authors contributed to the article and approved the submitted version.
